# A proof-of-concept application of gene set enrichment analysis principles to olfactory phenotyping in Parkinson’s disease

**DOI:** 10.3389/fnins.2026.1931818

**Published:** 2026-09-03

**Authors:** Jakub Šofranko, Natalia Hunarova, Michaela Škorvanová, Martin Kolisek

**Affiliations:** 1 Biomedical Centre Martin, Jessenius Faculty of Medicine in Martin, Comenius University in Bratislava, Martin, Slovakia; 2 Department of Medical Biochemistry, Jessenius Faculty of Medicine in Martin, Comenius University in Bratislava, Martin, Slovakia

**Keywords:** GSEA, neurodegeneration, olfactory, Parkinson’s disease, UPSIT

## Abstract

**Introduction:**

While gene set enrichment analysis (GSEA) framework is widely used on transcriptomics or proteomics data, its applications beyond “omics” data has not been extensively explored. Its principal nature enables it to apply to any dataset where variables can be rationally sorted into categories, or sets. This study demonstrates a novel application of GSEA outside of its traditional use by applying the framework to clinical olfactory phenotyping data.

**Methods:**

We applied the GSEA framework to University of Pennsylvania Smell Identification Test (UPSIT) results from Parkinson’s disease patients and healthy controls. Odors were classified into seven distinct functional categories, and a rank-based running sum was used to identify coordinated changes across those categories.

**Results:**

This approach revealed an exploratory pattern of negative enrichment within the “citrus/fresh” odor category in Parkinson’s disease patients. The computational pipeline was systematically evaluated alongside synthetic positive, negative, and permutation-based sanity checks.

**Discussion:**

Category-specific patterns of olfactory loss may offer additional insight into neurodegenerative processes, suggesting potential value for more refined olfactory phenotyping in Parkinson’s disease. This proof-of-concept highlights how established genomic algorithms can be directly adapted to clinical tools, questionnaires, or cognitive data where variables can be meaningfully grouped. However, the results here should be considered exploratory.

## Introduction

Interpreting or finding logic in complex data that can be categorized into pathways or biological processes is effectively achieved through Gene Set Enrichment Analysis (GSEA) (Subramanian et al., 2005). This approach operates by identifying coordinated changes within predefined sets of variables, rather than focusing on individual isolated components. This provides a robust framework for detecting systematic patterns that might otherwise remain hidden by biological or technical noise (Hung et al., 2012) and it has been applied widely in experimental context (Jin et al., 2023; Li et al., 2016). Although GSEA was developed for genomic data and has been widely used in transcriptomics and proteomics, this should not be its limitation. In recent years it has been transferred to the metabolomics data as well (Xia and Wishart, 2010). In theory, this framework can be used in any set of variables that can be meaningfully categorized, including olfactory domains. Analogous approaches have also been employed in neuroimaging, where brain regions are ranked by activation differences and tested against anatomical or functional atlases (Weinstein et al., 2023).

While Parkinson’s disease is mostly characterized by motor symptoms such as bradykinesia, rigidity or tremor, smell loss is also an established clinical biomarker (Mitchell et al., 2025; Postuma et al., 2015). Diagnosis is frequently conducted using University of Pennsylvania smell test (UPSIT) (Doty et al., 1984; Morley et al., 2018). Whether olfactory dysfunction reflects uniform impairment across odors or a selective vulnerability of specific odor categories remains debated, with studies reporting inconsistent findings regarding which odorants best discriminate patients with Parkinson’s disease from controls (Li et al., 2025; Mitchell et al., 2025). Given that odors can be grouped into functional categories, applying a GSEA-based framework offers a novel computational lens to explore whether coordinated sub-domain patterns can be detected in clinical olfactory datasets (Castro et al., 2013).

An advantage of GSEA is its widespread availability. Multiple platforms to perform GSEA exist, including R and python packages or standalone desktop applications. The framework can be readily applied with minimal adaptation (Fang et al., 2023; Korotkevich et al., 2021; Subramanian et al., 2007).

This paper aims to demonstrate the potential applications of GSEA outside of the traditional omics field, using olfactory phenotyping data in Parkinson’s disease as a model example.

## Methods

### Ethical statement

This study utilized fully anonymized, publicly archived repository data. Because the dataset was de-identified prior to public release and did not involve direct recruitment, interaction, or interventions with human subjects by the authors, this study was exempt from formal institutional bioethics committee review.

### Data collection and processing

To test the proposed framework, clinical olfactory phenotyping data were obtained from a publicly accessible repository Zenodo originating from a previously published clinical trial cohort - Ottawa Trial study (Li et al., 2024, 2025) (DOI: 10.5281/zenodo.13323913). The dataset includes complete University of Pennsylvania Smell Identification Test (UPSIT) scores for patients diagnosed with Parkinson’s disease (PD, *n* = 69) and age-matched healthy controls (HC, *n* = 118). The UPSIT consists of 40 distinct odorants presented in a scratch-and-sniff format.

For each odor, the identification rate was calculated separately for the PD and HC groups as the proportion of correctly identified responses within each group (i.e., number of correct identifications divided by the respective group size, *n* = 69 for PD and *n* = 118 for HC). The corresponding log_2_ fold change (FC) was then calculated as the ratio of these group-level identification rates (PD rate/HC rate). To examine the statistical significance of differences in odor identification between the two groups, a Fisher’s exact test was applied to 2 × 2 contingency tables, given the binary nature of the variables. Finally, a rank score (RS) for each odor was calculated for use in the gene set enrichment analysis (GSEA) according to the following formula:


RS=log(FC)2×(−log(p-value)10)

The extracted data along with the calculated variables for each odor are available in a Supplementary Table.

All 40 items were sorted in descending order based on their RS value to create the ranked item list.

As a descriptive measure of observed effect magnitude and statistical sensitivity, a *post hoc* power analysis was performed for each odor using Cohen’s h effect size, calculated from the PD and HC identification rates, with power estimated via a two-proportion test framework (α = 0.05; pwr package in R). Effect sizes ranged from *h* = −1.10 to *h* = −0.02 (mean *h* = −0.74). This corresponded to the descriptive mean power of 93% (median = 99.8%) across all 40 UPSIT items, with 36/40 items exceeding 80% power (Supplementary Table). These estimates are presented solely as descriptive metrics of observed effect magnitude and statistical sensitivity rather than formal evidence of adequate sample power. The R script is available on GitHub^1^.

Data were processed either in Microsoft Excel (v. 2408) or R (v. 4.6.0) using online environment^2^. Figures were created in R environment, exported and processed in Microsoft PowerPoint (v. 2408). Details on used libraries are in Supplementary materials.

### Classification of odors

To facilitate enrichment analysis, the 40 UPSIT odors were classified into seven functional categories: chemical/industrial, citrus/fresh, floral, fruity, savory food, spicy/woody, and sweet. Initial categorization was performed using the large language model Gemini 3.1 Flash-Lite (accessed June 2026) and subsequently reviewed and validated by the research team. The odor categories overlap with those identified in a relevant independent study, which derived discrete perceptual odor categories based on non-negative matrix factorization applied to a large-scale semantic odor profile database (Castro et al., 2013). The full distribution of odors across these categories is provided in Supplementary Data Sheet 1 (Table S1).

### Gene set enrichment analysis

Gene set enrichment analysis was performed in R using the *fgsea* package with a pre-ranked list of odor RS values and our defined categories (Korotkevich et al., 2021). Minimum category size was set to 3. Adaptive multilevel splitting approach was used to estimate *p*-values. Adjusting for multiple testing was done using the Benjamini-Hochberg method. FDR < 0.25 was considered significant. A fixed random seed was used to ensure reproducibility. The script and necessary files are available online on GitHub and the repository, respectively (Šofranko et al., 2026).

### Sanity check

Two sanity checks were performed. First, positive and negative control categories were manually constructed to represent expected significant and non-significant results, respectively. Second, a permutation test was conducted by randomly reassigning RS values across odors across 5,000 iterations to confirm that the observed enrichment exceeded what would be expected by chance. The script is also available online.

### Binomial logistic regression

Binomial logistic regression was performed in Jamovi (v. 2.7.33) to assess the discriminative power of individual odor categories for PD vs. HC classification. Separate univariate models were constructed for each category to avoid multicollinearity. To evaluate whether specific odor categories carry predictive value beyond overall olfactory impairment, a normalized category score was calculated for each participant as the proportion of correctly identified odors within a category relative to their overall UPSIT identification rate:


$$S\,c\,o\,r\,e=\frac{\frac{n_{c\,o\,r\,r}}{n_{t\,o\,t}}}{\frac{U\,P\,S\,I\,T\,\_\,s\,c\,o\,r\,e}{40}}$$

where n_*corr*_ is the number of correctly identified odors in the category, n_*tot*_ is the total number of odors in the category, and UPSIT_score is the total score out of 40. Whole regression report is in the online repository (DOI: 10.17632/zrtbf9ghfs.2).

## Results

**FIGURE 1:**
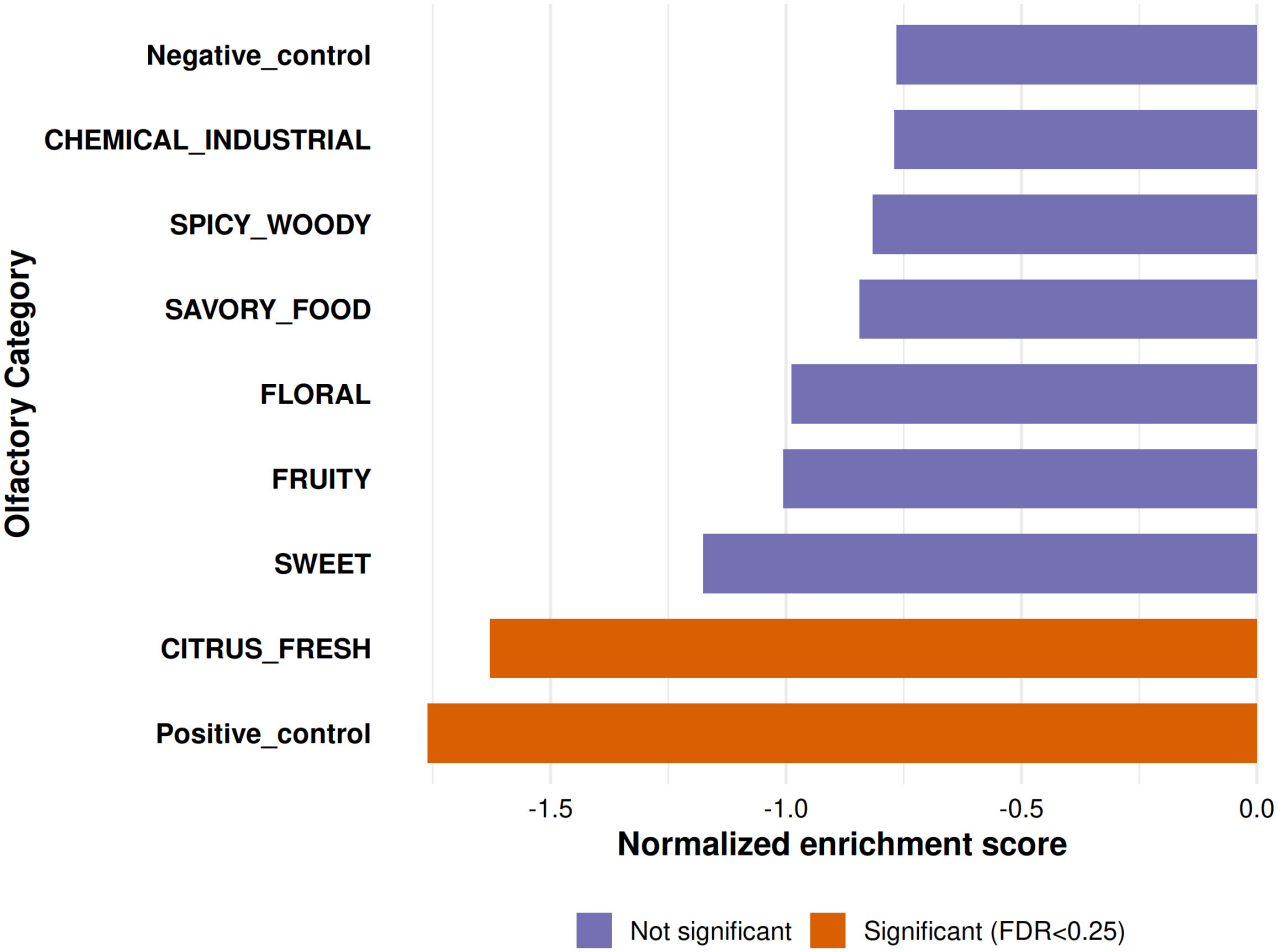
Gene set enrichment analysis (GSEA) analysis of odors’ categories. Normalized enrichment scores of olfactory categories based on UPSIT score of patients with Parkinson’s disease compared to healthy controls. Significantly enriched categories were citrus/fresh and positive control. Color legend is depicted in the figure.

**FIGURE 2:**
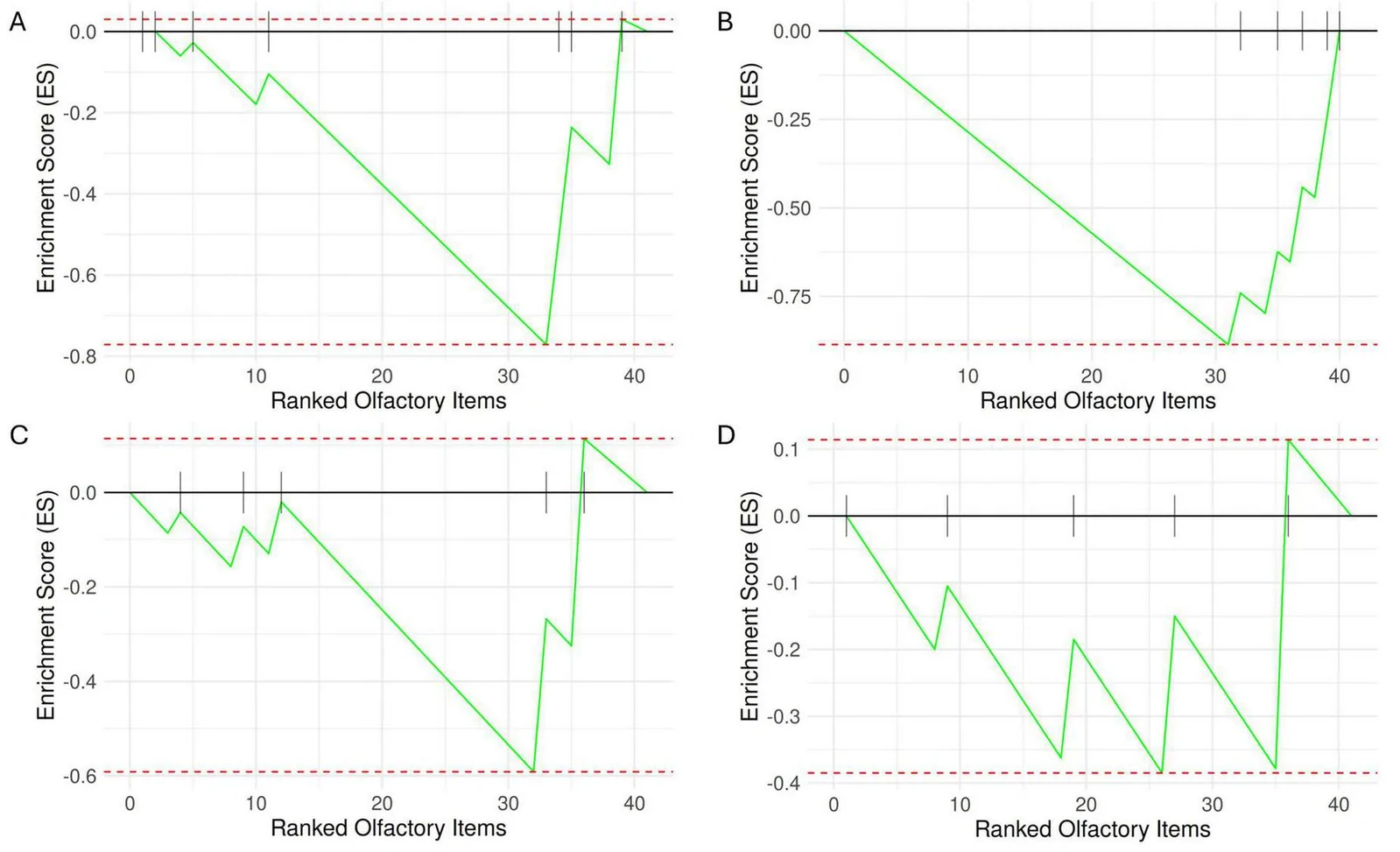
Running enrichment score plots. Rank-based category enrichment analysis profiles across standardized odor categories. The green running-sum curves illustrate the enrichment score (ES) walks across a 40-item ranked vector of olfactory performance. Horizontal black line marks denote the positional hits of specific categorical odor sets within the ranked list. Vertical black lines show zero ES. Horizontal red dashed lines represent the maximal, positive or negative, ES. Depicted are selected odor categories: the one that was significantly different between groups of patients – citrus **(A)**, positive control **(B)**, non-significant sub domain– sweet **(C)**, and negative control **(D)**.

Data obtained from UPSIT were used to construct odor categories along with the calculated and assigned RS for each odor to run GSEA. PD group was compared to HC to identify differentially enriched categories. From all the odor categories, except for positive control, “citrus/fresh” was significantly different between the groups and reached NES of −1.63 and *p*-adjusted 0.04 [Figure 1 and Supplementary Data Sheet 1 (Table S2)]. Positive control yielded NES −1.76 and *p*-adjusted 0.01. Also as expected, negative control was not significant with the NES −0.77 and *p*-adjusted 0.81. These sanity checks support the technical plausibility of the original findings and indicate that they were not artifacts of our pipeline. Running enrichment score plots for “citrus/fresh” category and positive control show similar pattern of specific odor hits, where we observe coordinated changes of odors (Figures 2A, B). In contrast, selected non-significant category “sweet” and negative control have their odors spread along the *x*-axis, meaning that there are no coordinated changes (Figures 2C, D). Taken together, these observations demonstrate the technical plausibility of our findings and ensure that our results are not merely artifacts of the pipeline. Even though we obtained similar results by employing GSEA desktop software, with the “citrus/fresh” category obtaining *p*-adjusted 0.039, these results should be interpreted as exploratory [Supplementary Figures S1–S4 and Supplementary Data Sheet 1 (Table S3)].

**FIGURE 3:**
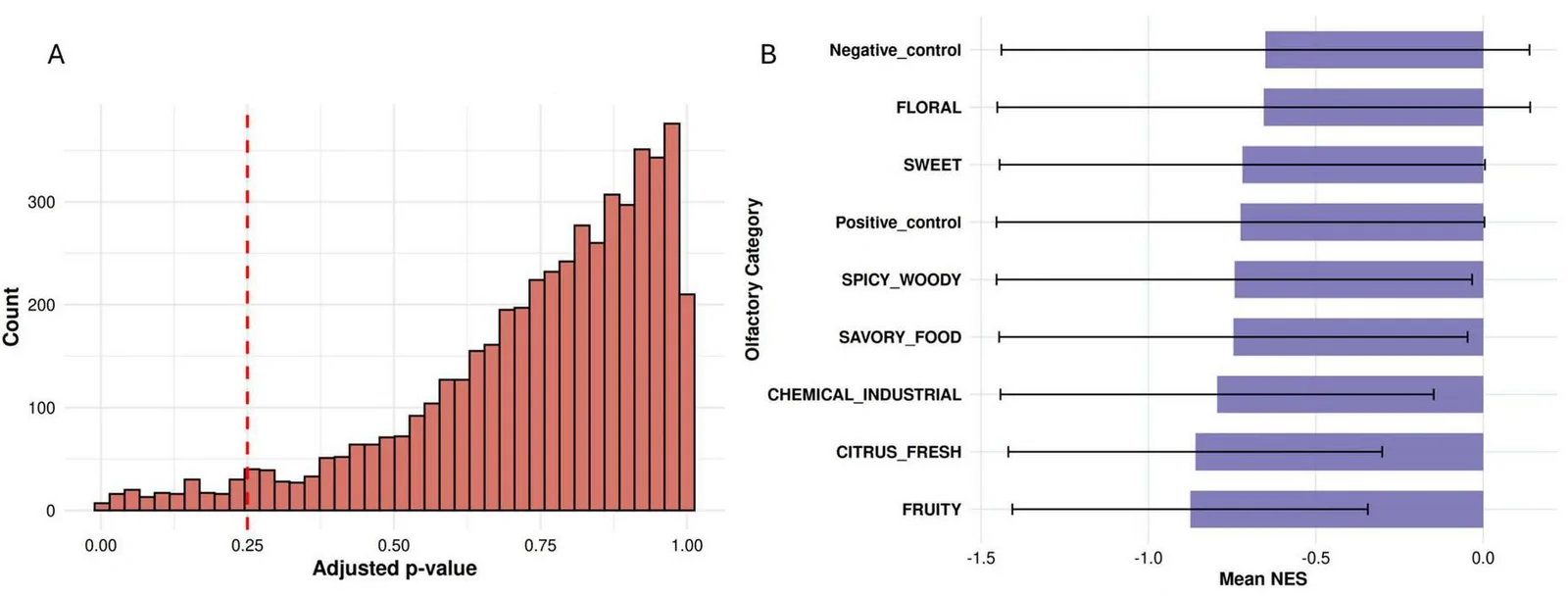
Permutation test simulation of GSEA analysis. Distribution of adjusted *p*-values for the “citrus/fresh” category based on 5,000 Monte Carlo simulations of GSEA analysis **(A)**. Vertical dashed red line marks significance threshold, which is *p*-adjusted < 0.25. Mean normalized enrichment scores (NES) for all of the odor categories with its standard deviation after iterations **(B)**.

Next, we generated a null distribution by randomly shuffling the RS values across all odors 5,000 times and re-running the GSEA pipeline for each iteration. This permutation-based sanity checks confirmed that randomly assigned RS values rarely yielded significant enrichment, with most resulting FDR-adjusted *p*-values exceeding 0.25 for “citrus/fresh” category, which was significant in previous analysis (Figure 3).

**FIGURE 4:**
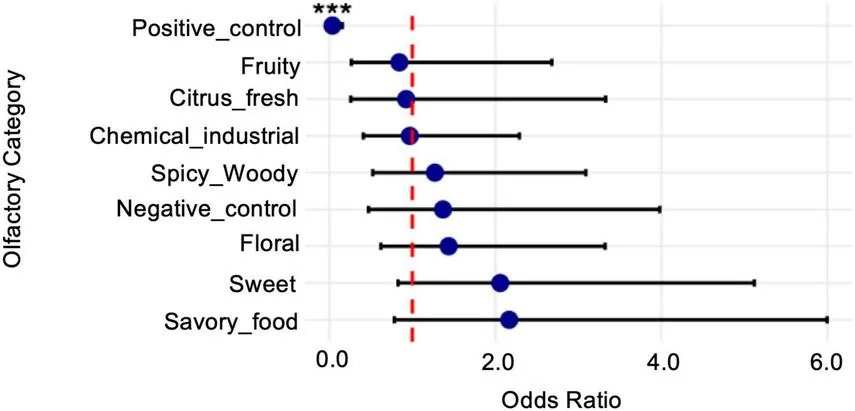
Forest plot of the binomial logistic regression. The plot displays the odds ratios (OR) for each odor category along with their 95% confidence intervals. The vertical dashed red line depicts the line of no effect (OR = 1). Three asterisks (***) indicate statistical significance at *p* < 0.001.

Finally, we employed binomial logistic regression to compare the performance in individual odor categories between the PD and HC groups. The only statistically significant result emerged from our designed category - positive control (*p* < 0.001; OR = 0.039, CI 95% [0.009, 0.157]). Negative control showed no significant discrimination power. Interestingly, the “Citrus/Fresh” category, which had previously demonstrated significance in the GSEA analysis, did not reach statistical significance within the logistic regression framework (Figure 4).

## Discussion

This proof-of-concept study demonstrates that the principles of Gene Set Enrichment Analysis (GSEA), originally engineered for genomics, could be adapted to clinical olfactory phenotyping data. By treating individual odor items as “genes” and sensory classifications as “gene sets,” the pipeline suggested a coordinated, though modest, impairment signal within the “citrus/fresh” odor category among patients with Parkinson’s disease. This raises the hypothesis that olfactory loss in PD may be oriented toward specific odor qualities rather than occurring uniformly (Daum et al., 2000; Double et al., 2003; Hähner et al., 2013).

Logistic regression also identified that our positive control category was significant, while “citrus/fresh” did not reach significance in this particular analysis. Logistic regression gives every item in a category the same weight when calculating the aggregate score, whereas GSEA weights each item according to its RS – so odors with a stronger PD–HC difference naturally have more influence on the final result. This matters here because several odors within the “citrus/fresh” category showed almost no difference between groups on their own (Supplementary Table), while a few others showed a clear effect. Consequently, an aggregate score may dilute localized effects within the category, whereas the rank-weighted nature of GSEA can detect coordinated but heterogeneous patterns. The fact that the positive control category was significant in both approaches suggests that when the underlying effect is sufficiently strong and consistent across items, both methods converge; this raises the possibility that the sensitivity of the two approaches could be systematically benchmarked against one another in future work. The significant result of the “citrus/fresh” category should be however, interpreted as an exploratory and hypothesis-generating result, rather than confirmatory, given the logistic regression has not supported the result and requires validation in an independent, larger dataset. Nevertheless, the combination of positive and negative controls, permutation-based test, and complementary logistic regression provides supporting evidence that the proposed framework is technically feasible and produces results that warrant further investigation.

Furthermore, the threshold for significance (*p*-adjusted < 0.25) as recommended by original GSEA paper (Subramanian et al., 2005) could be considered, since the high threshold could lead to false-positive findings. Consequently, results obtained using this threshold should be regarded as exploratory and hypothesis-generating rather than confirmatory, particularly when proposing potential biomarkers. On the other hand, results obtained under the stricter threshold would provide stronger evidence. To mitigate the possibility of false-positive findings associated with this threshold, we complemented the enrichment analysis with positive and negative controls, permutation-based simulations, and an independent analytical approach (logistic regression). While these analyses do not replace external validation, they provide additional support for the observed enrichment results.

As olfactory dysfunction often precedes motor symptoms by several years, identifying category-specific patterns of odor loss could contribute to refining early, non-invasive screening approaches for PD. Beyond indicating overall olfactory impairment, this category-based approach can help characterize which qualitative dimensions of smell are most affected, information not captured by a single aggregate UPSIT score. This could in theory serve as complementary information in the diagnostics or monitoring of the disease. Also, because distinct odorants engage different olfactory receptor families and downstream pathways, mapping category-level impairment could offer valuable insights into the underlying mechanisms and progression of the disease (Sharma et al., 2021). Moreover, this idea of GSEA repurposing should not be limited to olfactory data, but extended to other areas where multiple domains are assessed, such as quality-of-life questionnaires or cognitive assessments – tools commonly used to monitor neurodegeneration progression or in diagnosis – to identify individuals with subtle cognitive decline and observe which interacting domains of life are affected (Briceño et al., 2026; Islam et al., 2023; Jost et al., 2024). In general, the framework could be useful for any datasets, where individual variables could be meaningfully grouped into categories.

Although this study utilized data from the Ottawa Trial as a proof-of-concept model, the clinical significance of this category-specific olfactory loss requires validation in larger, independent, and longitudinal cohorts. Also, the sorting of the odors to the categories could be subjective, hence we opted to use LLM to categorize odors, to ensure reproducibility, with the final validation by authors. While traditional GSEA was optimized for large-scale data, we utilized the *fgsea* multilevel algorithm, which is specifically designed to calculate precise empirical *p*-values through item-ranking permutations, making it applicable to smaller sets. The methodological approach demonstrates an application of GSEA beyond its original scope, rather than a validated clinical tool. Hence, some other methodological parameters were not addressed here, due to exploratory nature of this communication. It is important to appropriately address choice of statistical test and RS calculation, or even make some optimization regardless of set size, number of sets, or number of permutations when applying it to specific problems.

Use of GSEA in this application is rationalized, because this framework is capable to detect subtle but coordinated changes, in contrast to other methods that require larger effect sizes. Additionally, as GSEA is a well-established framework, no new algorithms need to be developed – existing tools can be directly adapted for this purpose.

## Study limitations and future directions

A primary limitation of this proof-of-concept study is the reliance on a generative artificial intelligence model (Gemini 3.1 Flash-Lite) for the initial semantic and chemical categorization of odors. While AI classification provides a reproducible and objective linguistic framework, it may not perfectly capture the complex, lived sensory realities or the cross-cultural variations in human odor perception. Future studies should validate these category sets using expert human panel consensuses or direct chemical structure clustering. Additionally, while our sample size was sufficient to prove the computational pipeline works, validating this method across larger, longitudinal cohorts (such as the Parkinson’s Progression Markers Initiative) will be necessary to determine if specific category enrichment profiles can predict disease progression or track cognitive decline.

## Conclusion

In conclusion, adapting genomic algorithms like GSEA to clinical datasets opens a promising avenue for data repurposing. This framework is highly flexible and could easily be extended to other structured clinical tools, such as neuropsychological batteries, motor symptom scales, or lifestyle questionnaires. By looking at coordinated patterns rather than isolated items, clinicians and researchers can extract deeper, system-level insights from data that has already been collected. While early detection remains a priority in Parkinson’s disease research, the sub-domain enrichment observed here represents an analytical proof-of-concept rather than a validated clinical screening tool.

## Data Availability

The original contributions presented in this study are included in this article/Supplementary material, further inquiries can be directed to the corresponding authors.

## Footnotes

1
https://github.com/MitoLab-JLF/GSEA_olfactory.git

2
https://posit.cloud
