# Supplementary material for: A proof-of-concept application of gene set enrichment analysis principles to olfactory phenotyping in Parkinson’s disease
Source: Front Neurosci. 2026 Sep 3;20:1931818. doi: 10.3389/fnins.2026.1931818 (PMC13582382; doi:10.3389/fnins.2026.1931818)
Supplement: Supplementary file 1 [file Data_Sheet_1.docx]

**A Proof-of-Concept Application of Gene Set Enrichment Analysis Principles to Olfactory Phenotyping Data**

**Jakub Šofranko^1,*^, Natália Huňarová^2^, Michaela Škorvanová^1^, Martin Kolisek^1,*^**

^1^Biomedical Centre Martin, Jessenius Faculty of Medicine in Martin, Comenius University in Bratislava, Martin, Slovak Republic

^2^Department of Medical Biochemistry, Jessenius Faculty of Medicine in Martin, Comenius University in Bratislava, Malá Hora 4D, 036 01 Martin, Slovak Republic

*** Correspondence:**
Martin Kolisek
[martin.kolisek@uniba.sk](mailto:martin.kolisek@uniba.sk)

Jakub Šofranko

[jakub.sofranko@uniba.sk](mailto:jakub.sofranko@uniba.sk)

# 1 Supplementary figures and tables

## 1.1 Supplementary figures

**Supplementary Figure S1.** **Running enrichment score plot for ‘Citrus/Fresh’ odor category.** The green running-sum curve illustrates the enrichment score (ES) walks across a 40-item ranked vector of olfactory performance. Vertical black tick marks denote the positional hits of specific categorical odor sets within the ranked list. The horizontal grey line indicates zero ES.

**Supplementary Figure S2.** **Running enrichment score plot for Positive control category.** The green running-sum curve illustrates the enrichment score (ES) walks across a 40-item ranked vector of olfactory performance. Vertical black tick marks denote the positional hits of specific categorical odor sets within the ranked list. The horizontal grey line indicates zero ES.

**Supplementary Figure S3.** **Running enrichment score plot for ‘Sweet’ odor category.** The green running-sum curve illustrates the enrichment score (ES) walks across a 40-item ranked vector of olfactory performance. Vertical black tick marks denote the positional hits of specific categorical odor sets within the ranked list. The horizontal grey line indicates zero ES.

**Supplementary Figure S4.** **Running enrichment score plot for Negative control category.** The green running-sum curve illustrates the enrichment score (ES) walks across a 40-item ranked vector of olfactory performance. Vertical black tick marks denote the positional hits of specific categorical odor sets within the ranked list. The horizontal grey line indicates zero ES.

## 1.2 Supplementary Tables

**Supplementary Table S1.** Odors from University of Pennsylvania Smell Identification Test sorted to categories.

| **FRUITY** | **SWEET** | **CITRUS/ FRESH** | **SPICY/ WOODY** | **SAVORY FOOD** | **CHEMICAL/ INDUSTRIAL** | **FLORAL** | **Positive control** | **Negative control** |
| --- | --- | --- | --- | --- | --- | --- | --- | --- |
| Banana | Bubble_gum | Grass | Cedar | Cheddar_cheese | Gasoline | Leather | Rose | Root_beer |
| Cherry | Chocolate | Lemon | Cinnamon | Dill_pickle | Motor_oil | Lilac | Wintergreen | Strawberry |
| Fruit_punch | Coconut | Lime | Clove | Onion | Natural_gas | Rose | Licorice | Bubble_gum |
| Grape | Gingerbread | Menthol | Licorice | Peanut | Paint_thinner | Soap | Mint | Lemon |
| Peach | Root_beer | Mint | Pine | Pizza | Smoke |  | Motor_oil | Peach |
| Pineapple |  | Orange |  |  | Turpentine | |  |  |
| Strawberry | | Wintergreen | |  |  |  |  |  |
| Watermelon | |  |  |  |  |  |  |  |

**Supplementary Table S2.** Obtained variables for each odor category from GSEA analysis, such as *p*-value, adjusted *p*-value (*p*-adj), enrichment score (ES) and normalized enrichment score (NES) obtained from GSEA in R. Data are sorted by *p*-adj.

| **Odor category** | ***p*-value** | ***p*-adj** | **ES** | **NES** |
| --- | --- | --- | --- | --- |
| Positive control | 0.001 | 0.012 | -0.89 | -1.76 |
| Citrus/Fresh | 0.009 | 0.041 | -0.77 | -1.63 |
| Sweet | 0.234 | 0.703 | -0.59 | -1.18 |
| Chemical/Industrial | 0.812 | 0.812 | -0.37 | -0.77 |
| Floral | 0.503 | 0.812 | -0.52 | -0.99 |
| Fruity | 0.458 | 0.812 | -0.52 | -1.01 |
| Negative control | 0.811 | 0.812 | -0.38 | -0.77 |
| Savory Food | 0.728 | 0.812 | -0.42 | -0.84 |
| Spicy/Woody | 0.757 | 0.812 | -0.41 | -0.82 |

**Supplementary Table S3.** Obtained variables for each odor category from GSEA analysis, such as *p*-value, adjusted *p*-value (*p*-adj), enrichment score (ES) and normalized enrichment score (NES) obtained from desktop version of GSEA software. Data are sorted by *p*-adj.

| **Odor category** | ***p*-value** | ***p*-adj** | **ES** | **NES** |
| --- | --- | --- | --- | --- |
| Positive control | 0.002 | 0.02 | -0.89 | -1.73 |
| Citrus/Fresh | 0.011 | 0.039 | -0.77 | -1.64 |
| Sweet | 0.257 | 0.796 | -0.59 | -1.17 |
| Negative control | 0.828 | 0.823 | -0.38 | -0.75 |
| Chemical/Industrial | 0.798 | 0.901 | -0.37 | -0.77 |
| Floral | 0.53 | 0.930 | -0.52 | -0.97 |
| Spicy/Woody | 0.773 | 0.990 | -0.41 | -0.79 |
| Fruity | 0.478 | 1.000 | -0.46 | -1 |
| Savory Food | 0.732 | 1.000 | -0.42 | -0.83 |

# 2 R packages

data.table v. 1.18.4 (DOI: 10.32614/CRAN.package.data.table)

dplyr v 1.2.1 (DOI: 10.32614/CRAN.package.dplyr)

fgsea v. 1.38.0 (DOI: 10.18129/B9.bioc.fgsea)

pwr v. 1.3-0 (DOI: 10.32614/CRAN.package.pwr)
